# A multidisciplinary approach for the emergency care of patients with left ventricular assist devices: A practical guide

**DOI:** 10.3389/fcvm.2022.923544

**Published:** 2022-08-22

**Authors:** Matteo Cameli, Maria Concetta Pastore, Giulia Elena Mandoli, Federico Landra, Matteo Lisi, Luna Cavigli, Flavio D'Ascenzi, Marta Focardi, Chiara Carrucola, Aleksander Dokollari, Gianluigi Bisleri, Charilaos Tsioulpas, Sonia Bernazzali, Massimo Maccherini, Serafina Valente

**Affiliations:** ^1^Division of Cardiology, Department of Medical Biotechnologies, University of Siena, Siena, Italy; ^2^Division of Cardiology, Department of Cardiovascular Diseases -AUSL Romagna, Ospedale S. Maria delle Croci, Ravenna, Italy; ^3^Department of Cardiac Surgery, Cardiac Surgery, St. Michael Hospital, Toronto, ON, Canada; ^4^Department of Cardiac Surgery, Azienda Ospedaliera Universitaria Senese, Siena, Italy

**Keywords:** emergency, heart failure, urgency, LVAD (left ventricular assist device), mechanical circulatory support (MCS)

## Abstract

The use of a left ventricular assist device (LVAD) as a bridge-to-transplantation or destination therapy to support cardiac function in patients with end-stage heart failure (HF) is increasing in all developed countries. However, the expertise needed to implant and manage patients referred for LVAD treatment is limited to a few reference centers, which are often located far from the patient's home. Although patients undergoing LVAD implantation should be permanently referred to the LVAD center for the management and follow-up of the device also after implantation, they would refer to the local healthcare service for routine assistance and urgent health issues related to the device or generic devices. Therefore, every clinician, from a bigger to a smaller center, should be prepared to manage LVAD carriers and the possible risks associated with LVAD management. Particularly, emergency treatment of patients with LVAD differs slightly from conventional emergency protocols and requires specific knowledge and a multidisciplinary approach to avoid ineffective treatment or dangerous consequences. This review aims to provide a standard protocol for managing emergency and urgency in patients with LVAD, elucidating the role of each healthcare professional and emphasizing the importance of collaboration between the emergency department, in-hospital ward, and LVAD reference center, as well as algorithms designed to ensure timely, adequate, and effective treatment to patients with LVAD.

## Introduction

A left ventricular assist device (LVAD) is implanted to support cardiac function in patients with end-stage heart failure (HF), when the myocardium is no more capable of ensuring the necessary hemodynamic conditions to maintain normal vital functions. The device implantation stage is strictly a matter of cardiac surgeons and should take place only in designated centers under the coordination of the National Heart Transplantation Center.

Thanks to rapid improvements in technology using more reliable and durable devices and an increasing incidence of advanced HF, the use of LVAD is widespread in almost all developed countries (1–3); however, few centers have the required expertise and supplies. This entails the referral of many patients coming from the whole country to a reference LVAD center, even many miles away from the local center. After implantation, these patients will have to be permanently referred to the LVAD center for device management and follow-up; however, routine assistance will be demanded by their trusted cardiologists and family doctors. Moreover, in case of urgent health issues, whether device-related or generic, they would refer to the local healthcare service. The concept of “shared-care” was born to optimize the management of severe patients and patients with chronic diseases through improved communication between primary and specialty centers and knowledge sharing. The result is an improvement in the quality of care by primary care hospitals alongside a reduction in the workload for tertiary center and, therefore, the overall cost. It has also proven to be a feasible and effective strategy for patients with LVAD (4).

Therefore, as the number of patients treated with LVAD is increasing, many clinicians, from bigger to smaller centers and from cardiologists to all healthcare professionals, will have to deal with LVAD carriers in the near future, so they should be prepared for the possible risks associated with LVAD management. In fact, emergency treatment of patients with LVAD differs slightly from the conventional emergency protocols and requires specific knowledge to properly manage, avoid ineffective treatment, or provide dangerous consequences (5, 6).

Several authors have already addressed this concern, and a recent expert consensus has been published on this topic (7, 8) However, the importance of a multidisciplinary approach for these patients and cooperation between the emergency department (ED) and in-hospital ward, within the LVAD center or, for local centers, with the LVAD center, and the role of each healthcare professional in the critical management of these sensitive patients has not been fully elucidated.

The main purpose of this paper is to present a standard protocol addressed to all healthcare professionals, based on the available evidence and authors experience, focused on the role of multiple clinical parts for the care and management of patients with LVAD. This would provide algorithms designed to ensure an adequate and timely response of all clinicians involved in the emergency care areas and clear indications for the interaction between dedicated and non-dedicated professionals.

## LVAD-associated complications

Left ventricular assist device-associated complications may be classified into LVAD-specific and LVAD-related complications (9). LVAD-specific complications are those directly involving structural or functional properties of the device and include suction event, pump thrombosis, pump failure, pump stoppage, and driveline damage. Besides, complications referred to as “related” are those not directly affecting the device but due to its presence and associated treatment. LVAD-related complications are, therefore, bleeding, hemorrhagic or ischemic stroke, infections, right ventricular failure, dysrhythmia, and aortic regurgitation (10).

In most of the cases, LVAD-related complications present themselves as emergencies, requiring the patient to be admitted to the nearest ED. As reported by the Heart Failure Society of America (HFSA), the Society for Academic Emergency Medicine (SAEM), and the International Society for Heart and Lung Transplantation (ISHLT) in the 2019 consensus for managing emergencies in patients with ventricular assist devices (VAD) (1), among the most worrisome medical emergencies commonly reported in individuals with VADs are cardiac arrest, unstable arrhythmias, myocardial infarction, and unexplained hypotension.

The nearest healthcare center plays a crucial role in readily evaluating patients who arrive in an unstable condition and stabilizing their vital parameters before sending them to a center with appropriate expertise. Situations that typically require immediate transfer to a *primary VAD center are*

- cardiac tamponade,
- mechanical VAD failure,
- pump thrombosis,
- emergency non-cardiac surgery, and
- neurological events.

Many concerns about the management of such patients arise from their intrinsic precarious coagulation balance. In fact, LVAD carriers are both at high thrombotic and high bleeding risk. On one hand, the risk of thrombosis is mainly determined by the presence of a foreign body, a severely reduced ejection fraction, and possibly atrial fibrillation. On the other hand, bleeding risk is due to the assumption of vitamin K antagonists along with aspirin, the possibility of hepatic congestion consequent to right heart dysfunction, and von Willebrand acquired disease.

Table 1 lists specific recommendations for the primary assistance of the most common LVAD-specific and LVAD-related emergencies.

**Table 1:** Management of the main emergency conditions of patients with LVAD according to the Heart Failure Society of America (HFSA), the Society for Academic Emergency Medicine (SAEM), and the International Society for Heart and Lung Transplantation (ISHLT) consensus document (1).

**Clinical presentation**	**Recommendations**
Stroke (ischemic or hemorragic)	Ischemic -> endovascular treatment (call LVAD Center first)
	Hemorragic -> blood pressure control, discontinue or reverse anticoagulation, neurologist and neurosurgeon consultation
HF (*inadequate decompression of LV or RV failure)*	Diuretics
	Positive inotropic support (es.milrinone) for subacute/chronic right HF
Abdominal pain	Physical examination and assessment of medical history -> if urgent surgery is needed, send to LVAD center
Bleeding	Assess hemodynamic stability
	Stop source of bleeding (EGDS and/or colonscopy may be necessary
	Balance concomitant antithrombotic risk and the need for reversal agents (vitamin K, fresh frozen plasma, prothrombin complex concentrates)
	Transfusions (reduces rates of future heart transplantation)
*VAD-specific emergencies*	
Pump thrombosis	IV Heparin and consider surgery treatment (immediate transfer to LVAD center) or mechanical circulatory support (e.g., ECMO)
Pump stoppage or failure	Use ungrounded cable or place patients on batteries only *(less stable long-term choice)*
	Whenever pump stoppage of failure happens, immediate call LVAD center

## Emergency care

### Patient's approach

There are some pivotal indications that each healthcare worker should respect when dealing with LVAD carriers (Table 2).

- Undress the patient delicately and do not use sharp tools to remove clothes.
- Pay attention to device cables and batteries, and do not use potentially damaging tools (e.g., scissors and scalpel).
- Check that batteries are connected to cables and are correctly working.
- The cardiologist in charge of the coronary care unit (CCU) must be immediately notified of the patient's admission and should take charge of the patient.
- Contact the LVAD reference regional center (telephone number list in the CCU).
- Invite the parent with LVAD to participate (if they are absent, call them quickly).
- Make sure that the patient is provided with an extra battery.

**Table 2:** Primary practical indications for in-hospital emergency management of patients with LVAD.

**1. Immediate approach to LVAD patients in emergency/urgency clinical scenarios**. A) Undress the patient delicately and **Don't use sharp tools** to remove clothes. B) Pay attention to the **Cables** and **Batteries** of the device, avoiding the use of tools that could potentially damage them (e.g., scissors, scalpel). C) Check that batteries are **Connected** to cables and that are correctly **Working** D) The **Cardiologist** responsible of **CCU** must be immediately informed about the patient admission and should take charge of the LVAD patient. E) Inform the **LVAD reference regional Center** (telephone number list in CCU) F) Invite the PARENT TRAINED for LVAD use to participate (if he is absent, call him quickly). G) Be sure that the patient is provided with an **Extra-battery**.
**2. Evaluation of vital signs and physical examination**. A) Patient could present **No pulse** (the LVAD flow could be continue and not pulsating). B) Heart rate at ECG could differ from those evaluated with pulse if the device is continue-flow and is not synchronized with heart rate. C) **Pulse oxymeter** could be less accurate for the estimation of oxygen blood saturation. D) **Heart auscultation** is anomalous: heart sounds are partially concealed by the continuous LVAD noise. E) The absence of the continuos LVAD NOISE could indicate device dysfunction.
**3**. **The resuscitation maneuvres** could provoke **LVAD cannulas dislocation** (particularly, the cannulas positioned in LV apex and in aorta) leading to sudden death, therefore the use of these maneuvers is permitted only in extreme situation and, preferably, in presence of selected devices (Table 3) and should be applied only as the last chance after excluding other resolvable causes of circulatory arrest. Electrical cardioversion (ECV) and defibrillation are possible with any device, however, the aggressive treatment of arrhythmias in **asymptomatic patients should be avoided** (also in case of non-sustained ventricular tachycardia): in case of the performance of ECV or defibrillation, beware **not to place the metal plates in correspondence of the device**. All drugs listed in ACLS (Advanced Cardiac Life Support) could be administered.
**4. Blood pressure assessment** could be challenging for the absence of a pulsatile blood flow. Arterial blood pressures could be manually measured using the sphygmomanometer (also with Doppler assistance) with the first Korotkoff tone audible corresponding to **Medium Blood Pressure**, or alternatively, using an invasive system of blood pressure monitoring. A value of medium BP between 70 and 90 mmHg is considered the medium target in LVAD patients.
**5. Don't stop anticoagulation** therapy unless indicated by the CCU Cardiologist.
**6**. CCU nurse should be informed soon in order to check the availability of **beds in CCU**.
**7**. The patient must be **transferred to CCU** as soon as possible, unless the clinical conditions require an immediate treatment or an urgent transfer to LVAD center.

CCU, coronary care unit; LV, left ventricular; LVAD, left ventricular assist device.

Almost all patients with LVADs have a small tag on their controllers that indicate specific devices, the center of implantation, and an emergency phone number. It is paramount in an emergency to focus on the color of the tag, which could rapidly lead to recognize the type of device, since each color is paired with a specific device following the emergency medical services (EMS) guide (10). Currently, the most widely used devices are HeartMate III, Jarvik 2000, and Heartware. Even if different devices share some characteristics and possible management, there are significant differences from technical to practical aspects, which are fully presented in Table 3.

**Table 3:** Different LVAD device characteristics and subsequent different emergency management.

**Information/devices**	**Heart MATE 3 / II**	**Heartware**	**Jarvik 2000**
Mechanism	Centrifugal pump with full magnetic levitation of the rotor / axial continue–flow pump	Centrifugal flow pump	Axial continue–flow pump
Pulse	Normally absent or weak	Normally absent or weak	It could be present depending on myocardial contraction, preload, and afterload
Target vital signs	mBP 70–90 mmHg	mBP 75–90 mmHg (preferably use the doppler method to measure BP)	mBP 65–75 mmHg
Low–flow advices	Heart–shaped red light will appear with a continuous acoustic alarm	Triangular yellow light with acoustic alarm	Low–flow –> Light alarm Pump arrest –> stop signal with red bell and continuous acoustic alarm
Low–flow treatment	*Hypovolemia* –> fluid administration *Right HF* –> inotropes, fluids, hyperventilation	Evaluate if volume expansion is required	
Device flow velocity	Impossible to speed up in out–of–hospital environment	Impossible to speed up in out–of–hospital environment	Normally set on 3 velocity, it could be manually adjusted
Heparin therapy	Generally, not required (discuss with LVAD implantation center)	To decide whether to use heparin, contact LVAD implantation center	Generally, not necessary
Defibrillation	Possible	Possible (don't disconnect device before delivering current)	Possible (don't disconnect device before delivering current)
External or manual pump	Not present	Not present With ECM, high risk of device displacement: evaluate on clinical basis. If ECM has to be performed, evaluate pump function and position first	Not present ECM Possible
External pacing	Possible	Possible	Possible
External cables	One cable emerges from abdomen	One cable emerges from abdomen	One cable emerges from retro–auricular area or abdomen
Battery supply	Patient should have already been equipped with a set of black batteries (3 h duration) and gray batteries (14–17 / 8–10 h duration; charge conditions could be checked pressing the button on the battery cover) –>At least ONE cable must always be connected to a power generator: DON'T remove simultaneously the two batteries, otherwise the pump will stop	Device receive charge from one battery at a time: maximum duration 4–6 h (Both battery and controller have a light signal indicating charge status)	Only battery power source (not electrical current) 2 types of battery: Small and portable, 8–10 h duration (could be quantified pressing on the black button on the battery) Big supply battery, minimum 24 h duration

ECM, external cardiac massage; HF, heart failure; LVAD, left ventricular assist device; mBP, mean blood pressure.

### Clinical evaluation and advanced cardiovascular life support algorithm

**Figure 1:**
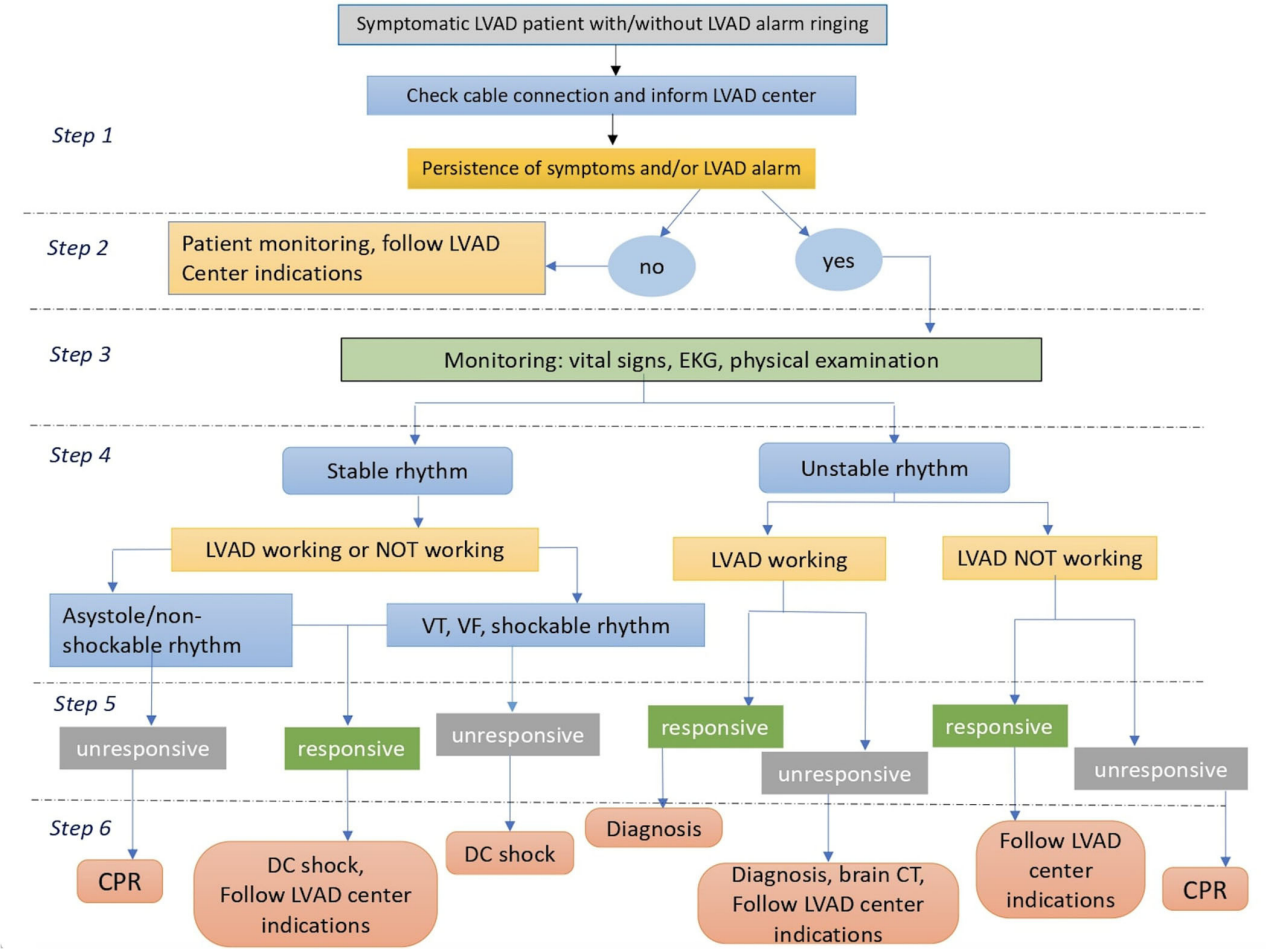
An algorithm for emergency management of patients with LVAD. CPR, cardiopulmonary resuscitation; DC, direct current; LVAD, left ventricular assist device; VF, ventricular fibrillation; VT, ventricular tachycardia; ED, emergency department.

Figure 1 shows a practical algorithm to be followed in case of emergency in patients with LVAD (11).

The healthcare worker conducting the clinical evaluation must remember that:

- The patient could have no pulse (LVAD flow could continue, therefore not pulsatile).
- Heart rate at electrocardiogram (ECG) could differ from those evaluated with a pulse if the device has a continuous flow and is not synchronized with the heart rate.
- Blood pressure (BP) assessment could be challenging in the absence of pulsatile blood flow. Arterial BP could be measured manually using the sphygmomanometer (also with Doppler assistance) with the first audible Korotkoff tone corresponding to the medium BP or using an invasive system of BP monitoring. A medium BP value between 70 and 90 mmHg is indicated.
- Pulse oximeter could be less accurate for estimating oxygen blood saturation.
- Heart auscultation is anomalous; heart sounds are partially concealed by continuous LVAD noise. The absence of continuous LVAD noise could indicate a device dysfunction.

Just after the clinical evaluation, the CCU nurse should be informed to check the availability of beds in the CCU. Remember not to stop anticoagulation therapy unless indicated by the CCU cardiologist. The patient should be transferred to the CCU as soon as possible, unless clinical conditions require immediate treatment or urgent transfer to an LVAD center.

If required, resuscitation maneuvers start immediately following the advanced cardiovascular life support (ACLS) LVAD algorithm. Remember that resuscitation maneuvers can provoke LVAD cannula displacement (particularly cannulas positioned at the apex of the left ventricle and in the aorta), leading to sudden death. Therefore, the use of these maneuvers is permitted only in extreme situations as a last chance after excluding other resolvable causes of circulatory arrest. Electrical cardioversion and defibrillation are possible with any device. When performing electrical cardioversion or defibrillation, care should be taken not to place metal plates in a position corresponding to the device. However, aggressive treatment of arrhythmias in asymptomatic patients should be avoided. All drugs listed in ACLS could be administered.

### Further assessments

**Figure 2:**
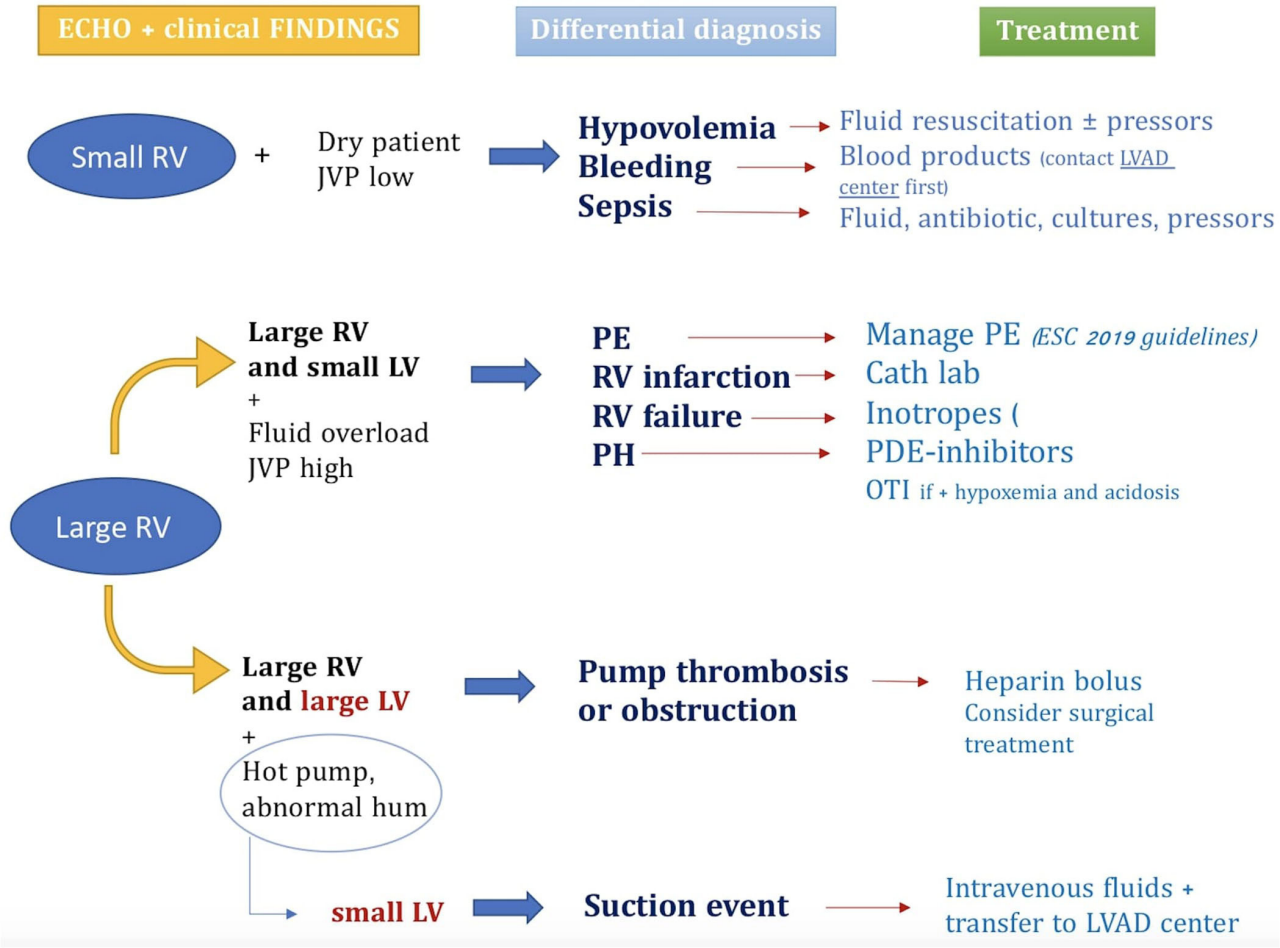
An algorithm for the differential diagnosis and treatment of LVAD emergencies that represent an important added value of performing bedside echocardiography. JVP, jugular venous pressure; LV, left ventricle; LVAD, left ventricle assist device; OTI, intubation orotracheal; PDE, phosphodiesterase; PE, pulmonary embolism; PH, pulmonary hypertension; RV right ventricle.

Additional assessments may be required to get to the root of the problem. These include:

a) Laboratory data: A complete blood count should be routinely performed, along with other assessments such as lactate dehydrogenase (LDH), haptoglobin, plasma free hemoglobin, troponin, and brain natriuretic peptide (BNP/N-terminal-pro-BNP) coagulation panel. Anemia may be indicative of ongoing bleeding or hemolysis if accompanied by an increase in LDH and free hemoglobin and a decrease in haptoglobin levels. Of note, hemolysis is often due to pump thrombosis in LVAD carriers, so it should be carefully excluded (12–15). On the other hand, abnormalities in the coagulation panel may support a diagnosis of pump thrombosis, if clinically suspected. Also, BNP/NT-pro-BNP could be elevated in this case, as well as in case of device malfunction or a new onset right HF (16–20). Troponin elevation can be found in multiple scenarios and should be specifically requested in case of new onset angor, dyspnea, or new alterations on the ECG.
b) Arterial blood gas analysis: To assess the presence of acidemia. Please consider that arterial puncture will not be easy as usual because the pulse is often diminished and the patients are always on anticoagulation therapy.
c) Imaging:

▪ **Chest X-Ray**: Easily available and helpful to evaluate pump and inflow–outflow cannula position (11).
▪ **Echocardiography**: Important to analyze pump flow, possible thrombosis, mechanical complications, LV hemodynamic conditions and filling, and valvular regurgitation [aortic regurgitation can frequently occur in patients with continuous LVAD flow due to multiple factors such as LV unloading (9)]. If available, it is important to perform bedside echocardiography to help focus the diagnosis. Figure 2 shows an algorithm to speed up diagnosis and treatment of the emergence of LVAD starting from echocardiographic findings (15, 16, 21).
▪ **Computed tomography (CT)**: It could help evaluate areas not visible by echocardiography, such as outflow pump cannula position, and the lack of information due to the poor acoustic window in these patients (22, 23). Moreover, cranial acquisition is crucial in case of suspected stroke, to differentiate hemorrhagic from ischemic ones.

Remember that magnetic resonance imaging (MRI) is contraindicated in patients with LVAD.

## Roles and responsibilities

Each healthcare worker plays a specific role in emergency care as described above.

### Role of the medical director

a) To ensure the distribution of this document and the comprehension of its content with reference to all possible participants in the management of patients with LVAD.
b) To organize a systematic pathway between LVAD centers and the local hospital to identify all patients with old and new LVAD implantations possibly pertaining to that area.
A list of *LVAD carriers* should be placed in a dedicate folder and stored in the ED or CCU.

### Role of the HF and LVAD multidisciplinary team

A) To ensure continuous update of this document based on the newest evidence.
B) To supervise compliance and correct application of the following procedure when a patient with LVAD is referred to the local hospital.
C) To verify that each patients with LVAD has been correctly identified in the medical records and marked as an “LVAD carrier.”
D) To provide a promptly available list of all national LVAD reference centers (to be stored in the CCU).

### Role of the physician who is in charge of a patient with LVAD

A) Inform the CCU referral cardiologist of the admission of a patient with LVAD.
B) To ensure that the HF and LVAD multidisciplinary team has been informed of the admission of a patient with LVAD.
C) To apply the management algorithm reported in Figure 1.
D) To ensure that a phone contact of the closest LVAD reference center is present and that all information about emergency phone numbers, emergency instructions, and battery supply of the device is available to healthcare workers.
E) To ensure systematic follow-up of the patient after discharge [following existing models in other clinical settings (24, 25)] following the Hub–Spoke model in collaboration with the LVAD reference center.

### Role of the CCU team

A) If required, to draw up an appropriate treatment plan that integrates clinical and nursing roles for the care of patients with LVAD.
B) To facilitate a timely transfer and admission of the patient with LVAD from the ED to the CCU.
C) To ensure that this protocol is followed specifically in case of the admission of a patient with LVAD.
D) To inform the LVAD implantation center of the patient's hospitalization as soon as possible (using the phone contacts on the list stored in the CCU-point D in paragraph 2.2).

### Role of biomedical engineer

A) Be available for any technical consultation in case of a device malfunction that is not clearly identifiable.
B) Provide technical indications for solving the problem.

### Role of nurse care manager

A) Build up and lead the development of a comprehensive, individualized care plan for each hospitalized patient.
B) Contribute to the education of patients and their families by having them fully understand their clinical condition, manage the symptoms associated with their condition, and understand treatment alternatives.
C) Address nonclinical issues that impact quality of life and outcome.

### Role of clinicians and nurses in the other departments in which patients with LVAD could be admitted (e.g., surgical department, neurologic department, and so on)

A) Inform the CCU referral cardiologist immediately (if not already done).
B) To ensure that each healthcare operator of the ward is aware of this document.
C) To monitor strict compliance with the advice contained in this document.

### Role of each healthcare professional

A) To be aware of the procedures explained in this document.
B) To perform them correctly in case of the admission of the patient with LVAD.

## Conclusions

The growing use of LVAD implantation as destination therapy worldwide has resulted in the need of specific training for clinicians of all specialties to manage patients with LVAD. Particularly, in the emergency setting, a standardized multidisciplinary approach and collaboration between small and VAD centers are essential to ensure the best treatment for patients. This document offers an easy consultation and practical guide for the appropriate management of emergencies in patients with LVAD.

## Author contributions

MC, MP, and SV had the idea for this paper. MC, MP, GM, and FL performed the literature search and analysis and drafted the manuscript. ML, LC, CT, MM, SB, FD'A, MF, and SV critically revised the work. All authors contributed to this review conception. All authors have read and approved the final manuscript.

## Conflict of interest

The authors declare that the research was conducted in the absence of any commercial or financial relationships that could be construed as a potential conflict of interest.

## Publisher's note

All claims expressed in this article are solely those of the authors and do not necessarily represent those of their affiliated organizations, or those of the publisher, the editors and the reviewers. Any product that may be evaluated in this article, or claim that may be made by its manufacturer, is not guaranteed or endorsed by the publisher.
